# Distinct host genetic determinants of HIV acquisition and disease progression: the case of classical *HLA* class I

**DOI:** 10.3389/fgene.2026.1982540

**Published:** 2026-10-09

**Authors:** Marion Amujal, James Bumba

**Affiliations:** Department of Immunology and Molecular Biology, College of Health Sciences, Makerere University, Kampala, Uganda

**Keywords:** elite controllers, highly exposed seronegative individuals, HIV acquisition, HIV host genetics, HIV susceptibility, host-pathogen interactions, long-term non-progressors, viral control

## Abstract

HIV acquisition and disease progression appear to be influenced by largely distinct host genetic factors. Whether infection becomes established is determined shortly after mucosal or parenteral exposure, before HIV-specific CD8^+^ T-cell responses become detectable. Progression is shaped by immune responses acting after infection is established and is strongly influenced by classical *HLA* class I variation. *CCR5Δ32* affects both phenotypes, raising the possibility of shared determinants, but evidence for genetic overlap remains limited. Here, we review evidence on whether classical *HLA* class I variation is associated with HIV acquisition across diverse exposure routes and study designs. A large collaborative genome-wide association study (GWAS) found no significant association between *HLA* class I variation and HIV acquisition after accounting for frailty bias, and candidate-gene associations have not been independently replicated. Future research should prioritize well-characterized exposure cohorts and sequencing approaches capable of detecting structural variation and resolving complex loci such as killer-cell immunoglobulin-like receptors (KIRs), particularly in underrepresented African populations.

## 1 Introduction

An estimated 40.9 million people were living with HIV in 2025, with 1.2 million new infections and 570,000 AIDS related deaths recorded that year (UNAIDS, 2026). HIV infection is characterized by marked heterogeneity in disease progression. In the absence of antiretroviral therapy (ART), some individuals progress rapidly to AIDS, whereas others maintain long-term control of viral replication and remain clinically stable for more than 10 years (Deeks and Walker, 2007). This variation is only partly explained by clinical and demographic factors, including age and comorbidities (Sabin and Lundgren, 2013) with a substantial contribution from genetic variation in both the host and the virus (Bartha et al., 2017). Host genetic variation accounts for approximately 8.4% of the variation in set-point viral load (spVL), while viral genetic diversity accounts for approximately 29% (Bartha et al., 2017).

Among host genetic factors, variation within the major histocompatibility complex (MHC), particularly classical *HLA* class I genes is consistently associated with HIV disease progression (McLaren et al., 2015; McLaren et al., 2023). HLA class I molecules present viral peptides to CD8^+^ T-cells, facilitating elimination of virus infected cells (Musey et al., 1997). Candidate gene studies first associated HLA-B*57 and HLA-B*27 with delayed progression (Kaslow et al., 1996) whereas HLA-B*35-Px subtypes were associated with accelerated progression (Gao et al., 2001). Subsequent hypothesis-free analyses confirmed the MHC as a key host locus: a GWAS of 6,315 HIV-infected individuals identified the MHC on chromosome six and the chemokine receptor gene cluster on chromosome 3 as major determinants of spVL, together explaining 14.5% of the variability in spVL (McLaren et al., 2015). Fine-mapping further localized the MHC signal to five amino acid positions in *HLA-A* and *HLA-B*, with *HLA-B* position 97 showing the strongest association (McLaren et al., 2015), while the chromosome 3 signal mapped to rs1015164, which was later shown to modulate CCR5 expression through the long non-coding RNA CCR5AS (Kulkarni et al., 2019).

Whether the same genetic determinants influence HIV acquisition is less clear. Acquisition occurs within days of exposure, before HIV specific CD8^+^ T-cell responses become detectable (McMichael et al., 2010). The best established genetic determinant of acquisition instead acts at viral entry. Homozygosity for a 32-base-pair deletion in *CCR5* (*CCR5Δ32*) prevents functional CCR5 expression and confers near-complete protection against infection with R5-tropic viruses (Dean et al., 1979; Liu et al., 1996). This led to the development of therapeutic strategies, including CCR5 antagonists (Fätkenheuer et al., 2005) and allogeneic hematopoietic stem cell transplantation from *CCR5*Δ32 homozygous donors (Hütter et al., 2009). Individuals heterozygous for *CCR5Δ32* express reduced levels of functional CCR5 and have been associated with slower HIV disease progression (Dean et al., 1979; Huang et al., 1996). The deletion occurs at appreciable frequencies in populations of European ancestry (∼10% allele frequency) but is rare or absent in African populations (Martinson et al., 1997). Beyond *CCR5*, reproducible determinants of acquisition remain scarce. Variants in *CD101* and *UBE2V1*, identified in African populations, are associated with increased risk but have not been replicated independently (Mackelprang et al., 2017).

The observation that *CCR5Δ32* influences both HIV acquisition and disease progression raised the possibility that these phenotypes share common host genetic determinants (McLaren et al., 2013). However, larger studies have provided little evidence for overlap (Serrano-Rísquez et al., 2024; Vince et al., 2014). Several reported associations have subsequently been attributed to methodological biases or have failed to replicate (Amujal et al., 2026). Here we review the evidence for whether classical *HLA* class I influences HIV acquisition, across exposure routes and study designs. We examine why its well-established effects on disease progression have not translated into reproducible effects on acquisition and consider the implications for future genetic studies of HIV susceptibility and disease progression.

## 2 Acquisition and progression are biologically distinct processes

Most HIV acquisition occurs through sexual exposure at the genital or rectal mucosa. Whether infection becomes established depends on events during the eclipse phase, the approximately 10-day period between HIV transmission and the onset of detectable plasma viraemia. During this period, the virus initially replicates locally at the site of exposure before disseminating to draining lymphoid tissues (McMichael et al., 2010). Sexual transmission across the mucosal epithelial barrier is inefficient, and most exposures do not result in productive infection. The estimated per-act transmission risk is 0.08% for receptive and 0.04% for insertive penile-vaginal intercourse, compared with 92.5% following transfusion of contaminated blood (Patel et al., 2014). Disruption of the mucosal barrier through trauma or concurrent genital infection can further increase the likelihood of acquisition (Galvin and Cohen, 2004; Haaland et al., 2009). The availability of susceptible target cells and local innate immune responses may also influence whether infection becomes established (Haase, 2010). When transmission occurs, infection is often established by a single founder virus, which preferentially infects CD4^+^ CCR5^+^ T cells (McMichael et al., 2010; Gasper-Smit et al., 2008). Acquisition is therefore determined primarily by events occurring at or shortly after exposure (Figure 1).

**FIGURE 1:**
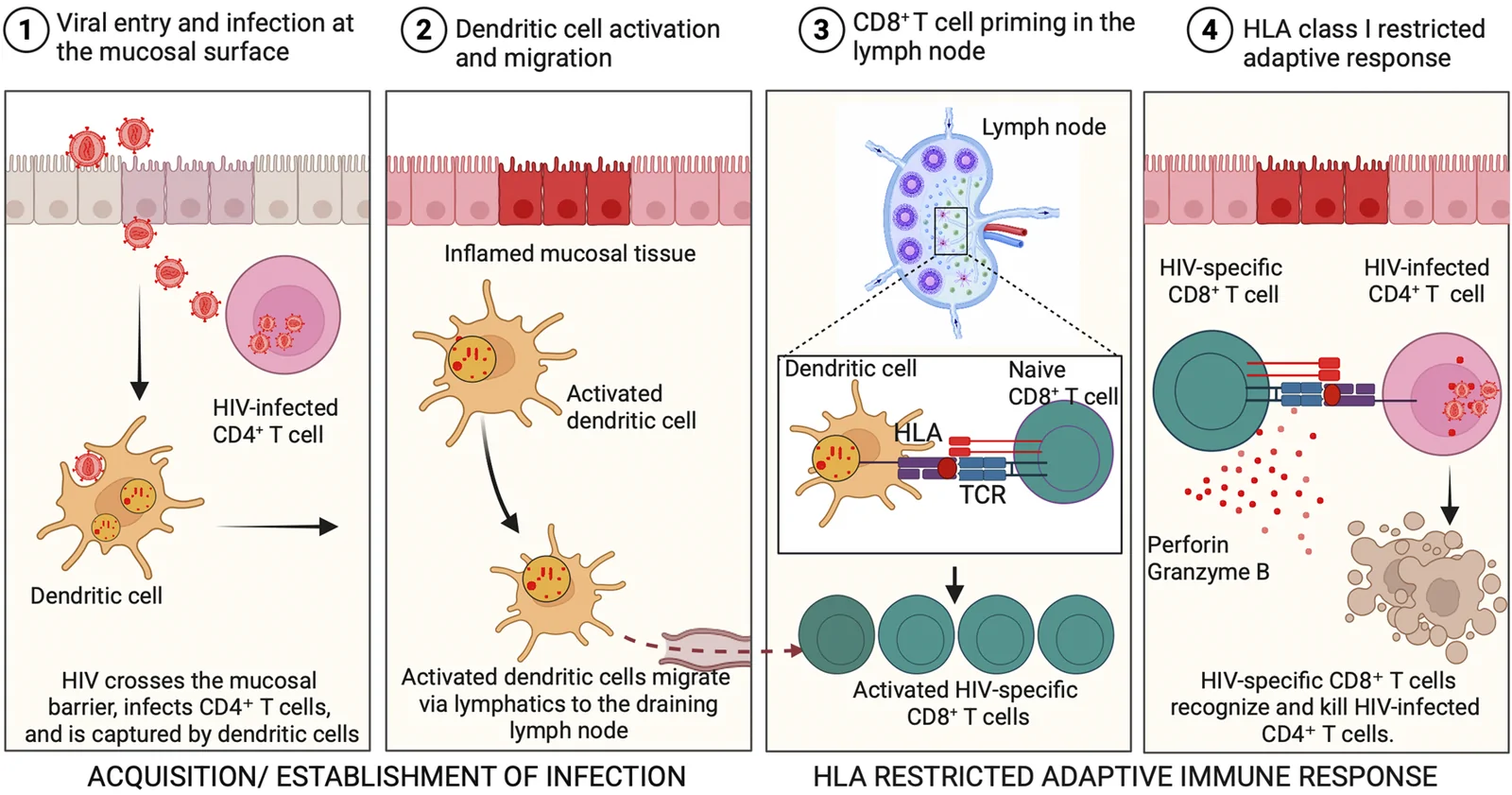
HIV acquisition and the development of HLA-restricted HIV-specific CD8^+^ T-cell responses. Following mucosal exposure, HIV crosses the epithelial barrier and infects susceptible CD4^+^ T cells, while viral antigen is captured by dendritic cells (UNAIDS, 2026). Activated dendritic cells migrate to draining lymph nodes (Deeks and Walker, 2007), where presentation of viral peptides by HLA class I primes naïve CD8^+^ T cells and promotes the expansion of HIV-specific CD8^+^ T cells (Sabin and Lundgren, 2013). These cells subsequently recognize and kill HIV-infected CD4^+^ T cells through cytotoxic mechanisms, including perforin and granzyme B release (Bartha et al., 2017). This sequence illustrates that HIV acquisition occurs before the development of the HLA-restricted HIV-specific CD8^+^ T-cell response that contributes to control of established infection.

Disease progression, in contrast, describes the deterioration of immune function after infection is established, commonly assessed by the rate of CD4^+^ T-cell decline (Vijayan et al., 2017). The virus disseminates to lymphoid tissues and replicates rapidly, resulting in peak viraemia (McMichael et al., 2010). HIV-specific CD8^+^ T-cell responses emerge around this peak and drive the subsequent decline in viral load (Goonetill et al., 2009). Viral load then stabilizes at a set point, with lower set-point viral load (spVL) indicating better viral control. Some CD8^+^ T-cell responses target functionally constrained epitopes, so escaping viruses replicate less efficiently, contributing to a lower spVL (Schneidewind et al., 2007). HLA-B*57:01 and B*27:05 are protective in European ancestry populations, and B*57:03 and B*58:01 confer protection in African populations (Goulder and Walker, 2012). However, because HLA-restricted CD8^+^ T-cell responses require priming and clonal expansion, they are unlikely to act within the interval in which acquisition occurs (McMichael et al., 2010). Moreover, HIV-specific CD8^+^ T-cell responses detected before seroconversion do not necessarily prevent acquisition. Women in the Nairobi sex-worker cohort with pre-existing responses subsequently seroconverted (Kaul et al., 2001), as did an exposed seronegative individual with broad CD8^+^ T-cell responses before infection (Liu et al., 2009). These observations suggest that HIV-specific CD8^+^ T-cell responses alone are insufficient to prevent acquisition.

## 3 *HLA* associations with HIV acquisition may reflect frailty bias

Frailty bias can arise when prevalent HIV-positive individuals are used as cases. Carriers of protective *HLA* alleles survive longer without treatment and may therefore become overrepresented among prevalent cases. This can create an apparent association with acquisition that instead reflects post-infection survival.

An early GWAS of HIV acquisition identified significant associations within the MHC in 6,334 HIV-infected individuals and 7,247 controls (McLaren et al., 2013). The lead variant, rs4418214, tags HLA-B*57:01 and HLA-B*27:05 (OR = 1.52, p = 3.6 × 10^−11^), two alleles with established roles in viral control and slower progression. The enrichment of these protective alleles among infected individuals initially appeared paradoxical. However, this association was driven disproportionately by cohorts enriched for slow progressors, in whom protective *HLA* alleles are overrepresented. When the analysis was restricted to 2,173 individuals with known seroconversion dates, the association with rs4418214 was markedly attenuated (p = 0.02). A sensitivity analysis showed that the weakened effect resulted specifically from reducing frailty bias, rather than from the reduced sample size. In contrast, *CCR5Δ32* homozygosity remained strongly associated with acquisition (*P* = 1 × 10^−6^) (McLaren et al., 2013).

A subsequent meta-analysis of 7,303 cases and 587,343 controls also identified a genome-wide significant *HLA* signal, with the lead variant rs41557415 located in *HLA-B* (McLaren et al., 2013; Duarte et al., 2022). However, because the McLaren dataset accounted for approximately 80% of the effective sample size, this signal was largely driven by the earlier dataset and therefore did not represent an independent replication (McLaren et al., 2013; Duarte et al., 2022). In addition, a transcriptome-wide association study (TWAS) conducted as part of the same analysis identified 15 susceptibility genes, with conditional analyses supporting roles for *HERC1*, *UEVLD*, and *HIST1H4K* in HIV acquisition (McLaren et al., 2013; Duarte et al., 2022).

## 4 HLA effects on HIV acquisition across exposure routes and transmission pairs

In studies of HIV acquisition, uninfected controls may never have been exposed to HIV, making cohorts with documented exposure particularly informative. Vince *et al.* investigated *HLA* class I and *KIR* variation in 325 HESN individuals with hemophilia A who received untreated plasma-derived factor VIII between 1979 and 1984 (Vince et al., 2014). More than 90% of patients given moderate or high-dose treatment during this period became infected, making the uninfected minority a well-defined HESN cohort. Controls were selected from the 1958 British Birth Cohort rather than from HIV-infected individuals, thereby minimizing frailty bias, and individuals homozygous for protective *CCR5* mutations were excluded to minimize confounding. No *HLA* allele, *KIR* gene, or *HLA-KIR* combination remained significant after multiple-testing correction. The authors attributed an initial association with HLA-B*08 to ancestry differences between the exposed cohort and the population controls. However, they cautioned against extrapolating these findings to other exposure settings, as *HLA* effects may differ by transmission route and exposure intensity (Vince et al., 2014).

However, the absence of an association is not limited to parenteral exposure. Serrano-Rísquez *et al.* examined 346 individuals with HIV and 363 HESN individuals of self-reported European ancestry from cohorts of men who have sex with men (MSM) and heterosexual serodiscordant couples in Spain, Italy, and the United States (Serrano-Rísquez et al., 2024). After correction for multiple testing, no *HLA-A*, *HLA-B*, or *HLA-C* allele was associated with HIV acquisition. Similarly, no KIR gene, KIR copy number variation, or *HLA-KIR* receptor-ligand combination showed significant associations. Thus, the lack of a reproducible association extends to sexual exposure, where the viral inoculum is generally far lower than in the haemophilia cohort (Serrano-Rísquez et al., 2024).

Transmission may also be influenced by the genetics of the infected source partner, not only the susceptibility of the exposed recipient. Serodiscordant couples allow these contributions to be separated, since both partners are genotyped. Gao *et al.* examined *HLA-B* alleles in 303 HIV infected men with haemophilia and their 323 exposed female partners, 43 of whom seroconverted (Gao et al., 2010). In the infected men, HLA-B*35 was associated with higher viral load and greater infectivity, whereas HLA-B*27 and HLA-B*57 were associated with lower viral load, although the reduction in infectivity was not significant. None of these alleles predicted seroconversion when carried by the exposed female partner (Gao et al., 2010). A similar pattern was reported in 429 serodiscordant couples in Zambia, where HLA-A*36:01 and HLA-C*18:01 in the index partner were associated with increased and reduced transmission, respectively, but showed no clear effect on acquisition when carried by the initially seronegative partner (Tang et al., 2008). These studies suggest that some *HLA* associations with HIV transmission may reflect effects on viral control in the source partner rather than direct effects on recipient susceptibility.

## 5 Reported *HLA* associations with HIV acquisition have not been independently replicated

Candidate gene studies in HESN cohorts initially suggested that specific *HLA* alleles might influence HIV acquisition. Peterson *et al.* genotyped *HLA* class I in more than 1,000 women from one such cohort in Nairobi and reported that A*01, C*06:02, and C*07:01 were associated with protection from seroconversion, whereas A*23:01, B*07:02, and B*42:01 were associated with rapid seroconversion (Peterson et al., 2013). Notably, the alleles protective against seroconversion were entirely distinct from those protective against progression in the same women, where A*74:01, B*14, and B*57:03 were associated with slower CD4^+^ decline (Peterson et al., 2013). The two outcomes showed different patterns within the same cohort, making population differences an unlikely explanation.

Luo et al. followed up two of these alleles with epitope characterization, choosing A*01:01 and B*07:02 because of their reported associations with resistance and susceptibility, respectively (Luo et al., 2012). A*01:01 presented three Gag epitopes compared with nineteen for B*07:02. Because HIV preferentially infects CD4^+^ T cells, the authors proposed that broader epitope presentation could expand the pool of susceptible target cells, whereas a narrower response might facilitate clearance of a small founder population without excessive immune activation. They also proposed that preventing a few founder viruses from establishing infection and controlling an infection already established are distinct immunological tasks (Luo et al., 2012).

A separate line of evidence suggests a potential role for NK cells in HIV acquisition. In a South African cohort of 224 HIV-infected mothers and 222 exposed infants, of whom 72 were infected and 150 remained uninfected, *KIR2DL3* with its *HLA-C1* ligand was associated with reduced infant acquisition (Paximadis et al., 2011). This association is consistent with a potential role for KIR-mediated NK-cell responses in acquisition and is of particular interest because HLA-C, unlike HLA-A and HLA-B, is not downregulated by HIV Nef (Cohen et al., 1999). However, the statistical support for these associations is limited as these studies were not corrected for multiple testing and have not been independently replicated (Peterson et al., 2013; Paximadis et al., 2011).

## 6 Innate and mucosal defenses as potential determinants of HIV acquisition

HIV-specific T-cell responses are undetectable in 30%–60% of HESN individuals across several cohorts (Tomescu et al., 2011; Miyazawa et al., 2009; Piacentini et al., 2008). The lack of reproducible *HLA* specific CD8 T cell responses with acquisition, raises the possibility that resistance may depend more strongly on innate and mucosal mechanisms.

NK-cell activity is regulated in part by interactions between HLA and KIR (Hölzemer et al., 2017). Increased NK-cell activity has been observed across HESN populations including injecting drug users, perinatally exposed infants, and serodiscordant couples (Tomescu et al., 2011; Scott-Algara et al., 2003). The *KIR*3DL1*h/*y genotype, carried with its HLA-B*57 ligand, was more frequent in HESN individuals than in those with primary infection (Boulet et al., 2008a). The activating receptor *KIR3DS1* was likewise enriched among HESN injecting drug users in Montreal (Boulet et al., 2008b). In Côte d’Ivoire, however, HESN female sex workers were enriched for inhibitory KIRs in the absence of their *HLA* ligands, including *KIR2DL2/KIR2DL3* without *HLA-C1* and *KIR3DL1* without *HLA-Bw4* (Jennes et al., 2006). The authors proposed that, without their cognate ligands, inhibitory KIRs cannot suppress NK-cell activity, allowing a faster response to infected cells at the point of exposure (Jennes et al., 2006). Associations have also been reported for non-classical *HLA* class I. In Zimbabwean women, HLA-E*0103 and the HLA-G*0105N null allele were associated with reduced infection risk (Lajoie et al., 2006), with both regulating NK-cell activity through CD94/NKG2A and ILT2/ILT4, respectively (Tomescu et al., 2011; Arns et al., 2020). These findings support a potential role for NK-cell mediated mechanisms in resistance to HIV acquisition (Figure 2).

**FIGURE 2:**
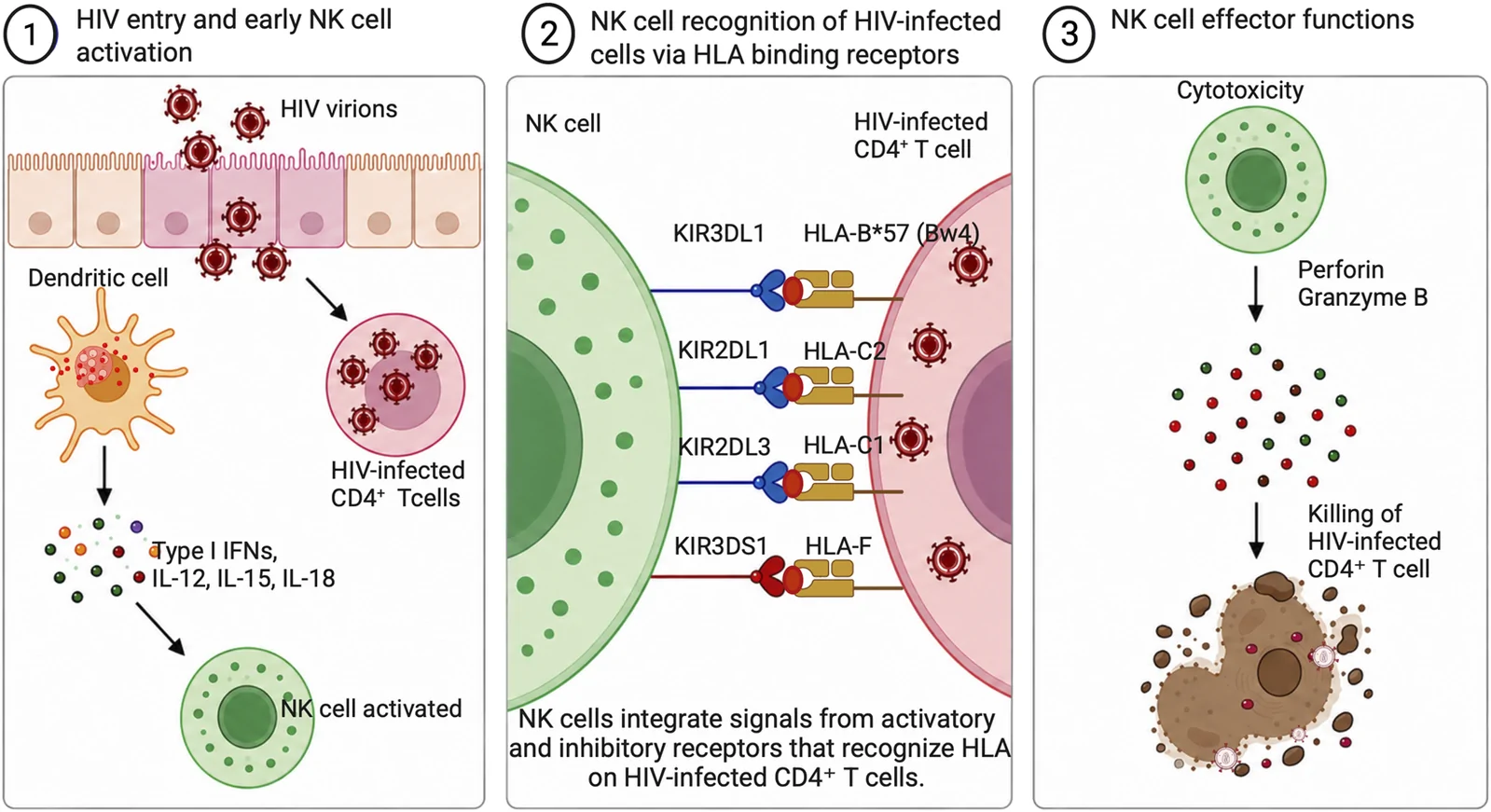
HLA-KIR interactions and NK-cell responses during early HIV exposure. Following HIV exposure, signals generated during the early host response promote NK-cell activation (UNAIDS, 2026). NK-cell activity is regulated by the balance of activating and inhibitory signals received through KIRs interacting with HLA ligands on HIV-infected cells (Deeks and Walker, 2007). These interactions can promote NK-cell cytotoxicity, including perforin and granzyme B release and the killing of infected CD4^+^ T cells (Sabin and Lundgren, 2013). HLA-KIR interactions therefore provide a potential mechanism through which host genetic variation could influence innate immune responses during the early stages of HIV exposure.

Innate defenses at the mucosal surface may provide another layer of protection. The β-chemokines MIP-1α, MIP-1β, and RANTES compete with HIV for the CCR5 coreceptor and inhibit entry (Amella et al., 2005), and elevated production has been reported in discordant couples, sex workers, and MSM (Furci et al., 1997; Hasselrot et al., 2010). In Kenyan sex workers, elevated cervicovaginal elafin/trappin-2 was associated with reduced HIV acquisition (Iqbal et al., 2009), while a separate proteomic study identified increased levels of the antiproteases Serpin A1, Serpin A3, Cystatin B, and A2ML1 in HIV-resistant women (Burgener et al., 2011). Increased expression of the intrinsic antiviral restriction factors MX2 and APOBEC3G has also been reported in HESN populations (Stein et al., 2015; Vázquez-Pérez et al., 2009). However, these associations have not been consistent, and cervicovaginal RANTES, lactoferrin, and SLPI concentrations may also be influenced by bacterial vaginosis and genital inflammation, complicating their interpretation (Novak et al., 2007). Overall, these observations support further investigation of innate and mucosal defenses that act early during HIV exposure. Key studies discussed throughout this review are summarized in Table 1.

**TABLE 1:** Key studies investigating host genetic variation in HIV acquisition.

Study	Study design/population	Sample size	Exposure route	Genetic variation investigated	Major findings	Key limitation
McLaren et al. (2013)	GWAS meta-analysis; European ancestry	6,334 PWH, 7,247 controls	Sexual and parenteral	Genome-wide common SNPs and indels	No common-variant association except *CCR5Δ32* homozygosity; the MHC signal was consistent with frailty bias	Prevalent cases were enriched for LTNP, introducing frailty bias; population controls had unknown HIV exposure
Duarte et al. (2022)	GWAS meta-analysis and TWAS; European ancestry	7,303 cases; 587,343 controls (effective N = 17,015)	Multiple exposure routes	5,347,926 genetic variants (GWAS) and genetically predicted gene expression (TWAS)	One genome-wide significant MHC locus, overlapping the McLaren et al. signal; TWAS implicated 15 genes, with *HERC1* the strongest candidate	HIV exposure was not characterized in population controls
Vince et al. (2014)	HESN with haemophilia A vs. population controls; European ancestry	325 HESN; 855–1,916 controls, depending on analysis	Parenteral; plasma-derived FVIII concentrates	*HLA* class I alleles, *KIR* genes and *HLA-KIR* combinations	No *HLA*, *KIR*, or *HLA*-*KIR* association after correction for population stratification and multiple testing	Findings from high parenteral exposure may not generalize to other exposure routes
Serrano-Rísquez et al. (2024)	HESN and PWH from MSM and heterosexual serodiscordant couples; European ancestry	346 PWH; 363 HESN	Sexual; MSM and heterosexual	*HLA-A*, *B* and *C*; *KIR* genes and copy number; *HLA-KIR* combinations	No *HLA*, *KIR*, or *HLA-KIR* association after correction for multiple testing	Limited power to detect effects of low-frequency alleles
Gao et al. (2010)	Transmission pairs: HIV-positive men with haemophilia and female partners; predominantly European American	303 men; 323 partners (43 seroconverted); 276 women HLA-B genotyped	Heterosexual; male-to-female	*HLA-B* alleles	Source *HLA-B* was associated with infectivity, viral load and disease progression; recipient HLA-B was not associated with acquisition	Small number of transmission events limited power to detect recipient *HLA-B* effects
Tang et al. (2008)	Targeted *HLA* association study; HIV-discordant couples, Zambia	429 couples (205 transmitting; 224 non-transmitting)	Heterosexual; male-to-female and female-to-male	*HLA-A*, *B* and *C* alleles and haplotypes	Index-partner *HLA* was associated with transmission and viral load; recipient *HLA* was not associated with acquisition	Recipient acquisition analyses were restricted to *HLA* identified in the transmission analysis
Peterson et al. (2013)	Targeted *HLA* association study; Pumwani Sex Worker Cohort, Nairobi, Kenya	338 seronegative women at enrolment (149 seroconverted)	Repeated sexual exposure; sex workers	*HLA-A*, *B* and *C* alleles	*HLA* alleles associated with seroconversion largely differed from those associated with disease progression	Associations were not corrected for multiple comparisons
Paximadis et al. (2011)	Targeted *KIR-HLA* association study; mother-infant cohorts, South Africa	224 mothers; 222 infants (72 infected; 150 exposed uninfected)	Mother-to-infant; predominantly intrauterine and intrapartum	*KIR* gene content and genotypes; *HLA-B* and *C* alleles/allotypes; *KIR-HLA* combinations	Infant *KIR2DL3* with *HLA-C1* was associated with reduced acquisition after adjustment for maternal viral load	Associations were not corrected for multiple comparisons
Mackelprang et al. (2017)	WGS extreme-phenotype discovery with replication; heterosexual serodiscordant couples, sub-Saharan Africa	Discovery: 100 (50 seroconverters; 50 HESN); replication: 261 (53 seroconversions)	Heterosexual; exposure quantified	Rare and intermediate-frequency functional variants in genic regions	*CD101* and *UBE2V1* variants were associated with increased acquisition risk; neither was associated with spVL	Association analysis was restricted to predicted functional variants in genic regions

HESN, HIV-exposed seronegative; KIR, killer-cell immunoglobulin-like receptor; MHC, major histocompatibility complex; MSM, men who have sex with men; PWH, people with HIV; SNP, single-nucleotide polymorphism; spVL, set-point viral load; TWAS, transcriptome-wide association study; WGS, whole-genome sequencing.

## 7 Discussion

Across GWAS and candidate-gene studies, classical *HLA* class I variation has not shown a reproducible association with HIV acquisition in either parenteral or sexual exposure (Serrano-Rísquez et al., 2024; Vince et al., 2014), unlike its well-established effects on disease progression (McLaren et al., 2015; McLaren et al., 2023). This is consistent with the different immune mechanisms operating during acquisition and established infection. Reported *HLA*-*KIR* associations suggest a possible NK-cell contribution, though no consistent receptor-ligand effect has emerged across populations.

Important gaps remain in the characterization of genetic variation beyond common SNPs. Structural variants, including copy-number variants, inversions, deletions and duplications, remain largely uncharacterized. Even where WGS has been applied, analyses have often been restricted to genic sites, leaving the non-coding genome underexplored. The WGS study in African serodiscordant couples nonetheless identified *CD101* and *UBE2V1* variants associated with acquisition but not with spVL (Mackelprang et al., 2017), further supporting distinct genetic determinants of acquisition and post-infection control. Complex loci present an additional challenge. *KIR*, for example, exhibits extensive gene-content and copy-number variation (Jiang et al., 2012), which is incompletely captured by standard SNP arrays. Direct sequencing of such loci, coupled with expression data, may reveal effects missed by conventional SNP-based approaches and exome sequencing.

Ancestry diversity is another important limitation of the current evidence. Large genetic studies of HIV acquisition have predominantly involved European ancestry populations**,** despite the high burden of HIV in Africa (UNAIDS, 2026). This is particularly relevant for highly polymorphic loci such as *HLA* and *KIR*, for which allele frequencies and haplotype structure vary substantially across populations and may limit the generalizability of findings across ancestries (Creary et al., 2021; Misra et al., 2018). Future studies should prioritize cohorts with well-characterized exposure and known seroconversion dates to reduce exposure misclassification and frailty bias. In transmission pairs, genotyping both partners can distinguish effects on source infectiousness from recipient susceptibility. However, effective pre-exposure prophylaxis (PrEP) increasingly complicates prospective studies of HIV acquisition, increasing the value of historical cohorts with stored specimens. Together, greater ancestry diversity, improved phenotype definition and more robust genomic approaches would help define the genetic determinants of HIV acquisition and clarify how it differs from that of post-infection viral control.
